# Barriers to and enablers of the use of the Otology Questionnaire Amsterdam in clinical practice—a qualitative post-implementation study

**DOI:** 10.1186/s41687-024-00741-9

**Published:** 2024-08-14

**Authors:** J.T. Kraak, K. Verhoef, S.E. Kramer, P. Merkus

**Affiliations:** Amsterdam UMC, Vrije Universiteit Amsterdam, Otolaryngology-Head and Neck Surgery, Ear and Hearing and Amsterdam Public Health Research Institute, de Boelelaan 1117, Amsterdam, The Netherlands

## Abstract

**Background:**

The Otology Questionnaire Amsterdam (OQUA) is developed to evaluate multiple ear complaints and their impact on patients’ daily lives. The current clinical use of this questionnaire is below the potential utilization.

**Aim:**

To identify the barriers and enablers of using the OQUA as perceived by ENT surgeons and patients and provide recommendations for an implementation strategy.

**Methods:**

Prospective and qualitative analysis was performed using focus groups and interviews with ENT professionals (n = 15) and patients (n = 25) with ear complaints of one tertiary referral hospital and two regional hospitals. Barriers and enablers were identified and classified by using the Capability-Opportunity-Motivation-Behavior model and the Theoretical Domains Framework. Suggestions for an implementation strategy will be made accordingly.

**Results:**

ENT professionals’ barriers included lack of knowledge and skills to use the OQUA, inadequate technological support and perceived time constraints during consultation, uncertainty about the clinical relevance and lack of feedback on the outcomes of the OQUA. Enablers included beneficial consequences of the OQUA for the professional, organization and science. Patients’ barriers included lack of knowledge about the objective and usefulness of the OQUA, perceived burden, difficulties in completing the questionnaire and insufficient feedback during consultation. Patient enablers included beliefs about beneficial consequences of the OQUA for the patient, health care and society. Suggested interventions involved education, training, environmental restructuring and incentivisation.

**Conclusion:**

Based on the findings, we propose an implementation strategy should focus on education and training about the objective, outcomes and relevance of the OQUA, environmental restructuring regarding the optimal use of the OQUA, and incentivisation with feedback on the valuable outcomes of the OQUA for the patient, professional and healthcare. Future research is needed to determine the feasibility of the implementation strategy.

**Supplementary Information:**

The online version contains supplementary material available at 10.1186/s41687-024-00741-9.

## Introduction

In the past few decades, recognition for providing patient-centred care has grown significantly, which has translated into the increasing development of patient-reported outcome measures (PROMs) [1, 2]. The use of these outcomes can lead to improving the patient-clinician relationship and increasing the patient’s participation in determining treatment [2, 3]. In addition, PROMs can be utilized to compare healthcare providers, settings, and patient groups, which may lead to improving the quality of healthcare [1]. However, before the potential benefits can be achieved, PROMs need to be successfully implemented in clinical settings.

The implementation of PROMs into routine care is known as a challenging process, as it requires a shift in the clinical practice and changes in the individual behaviour of healthcare providers and patients [4, 5]. Previous studies have demonstrated the above through capturing the limited uptake of PROMs in clinical practices [3, 6]. An essential part of Implementation science (IS) is the use of theories and frameworks, which can provide insight into why the implementation of an innovation succeeds or fails [7]. The core of these IS approaches is twofold: (1) identifying barriers to and enablers of the implementation of the innovation as experienced by individuals, providers, organizations, or other stakeholder groups and (2) developing and applying an implementation strategy that address the barriers and enablers [8]. Prior research has demonstrated that the IS approach is largely harmonious with the implementation of PROMs [3] and that their frameworks can be applied to improve clinical adoption [9].

The Otology Questionnaire Amsterdam (OQUA) has been implemented as part of standard care in in Amsterdam UMC by December 2020. It is developed to evaluate multiple ear complaints and their impact on patients’ daily lives [10]. The questionnaire contains 34 items and covers eight ear complaints (earache, pressure sensation, itching, tinnitus, hearing loss, ear discharge, loss of taste, dizziness) and their impact [11]. Supplementary Material S1 shows the validated version of the OQUA by Kraak, van Dam [11].

The OQUA is linked to all surgical otological interventions, in which the patient automatically receives an invitation to complete the questionnaire 2 weeks before and 3 and 12 months after intervention. Pre- and post-intervention scores are used to evaluate the effectiveness of the surgical intervention from the patient’s perspective. An example of the graphical representation of the OQUA is provided in Supplementary Material S2. In addition, the OQUA has been implemented by other hospitals, including Noordwest Ziekenhuisgroep (NWZ) and Deventer Hospital.

From December 2020 till December 2022, response rate on the OQUA was low. In a total of 288 patients who received the questionnaire, 15.5% completed the OQUA both pre- and post-operatively. The descriptive data analysis of this retro-perspective patient cohort is shown in Table 1.

**Table 1:** Quantitative descriptive analysis of the percentage of patients at Amsterdam UMC who completed the OQUA pre- and postoperatively from December 2020 to December 2022 (retro-perspective patient cohort)

	Patients (N)	Percentage (%)
Total	288	100
Active My Chart^a^	212	73.6
OQUA *pre-operative*	110	51.8
OQUA *post-operative*	33	15.5

^**a**^Required for obtaining access to the OQUA

The aim of this study was to identify the barriers to and enablers of using the OQUA as perceived by ENT surgeons and patients. Based on the identified barriers and enablers, the study will provide recommendations for the development of effective interventions as part of an implementation strategy. Previous research has demonstrated that enablers are dependent on the context and availability of local resources. Barriers in general include uncertainty about the relevance and negative consequences of PROMs, competing requirements in daily practice, and technological restrictions [3]. We hypothesize that the identified barriers in this study will be coherent with the existing literature [3, 6, 12, 13] and that the development of an implementation strategy is needed to overcome these barriers and enhance the enablers.

## Methods

A prospective, multicentre, qualitative study was performed at the ENT department of the Amsterdam UMC, NWZ and Deventer hospital Netherlands from November 2022 to April 2023. Focus groups and interviews were conducted with ENT professionals and patients to identify the barriers to and enablers of the use of the OQUA in clinical practice. The study was designed according to the Standards for Reporting Qualitative Research [14].

**Fig. 1:**
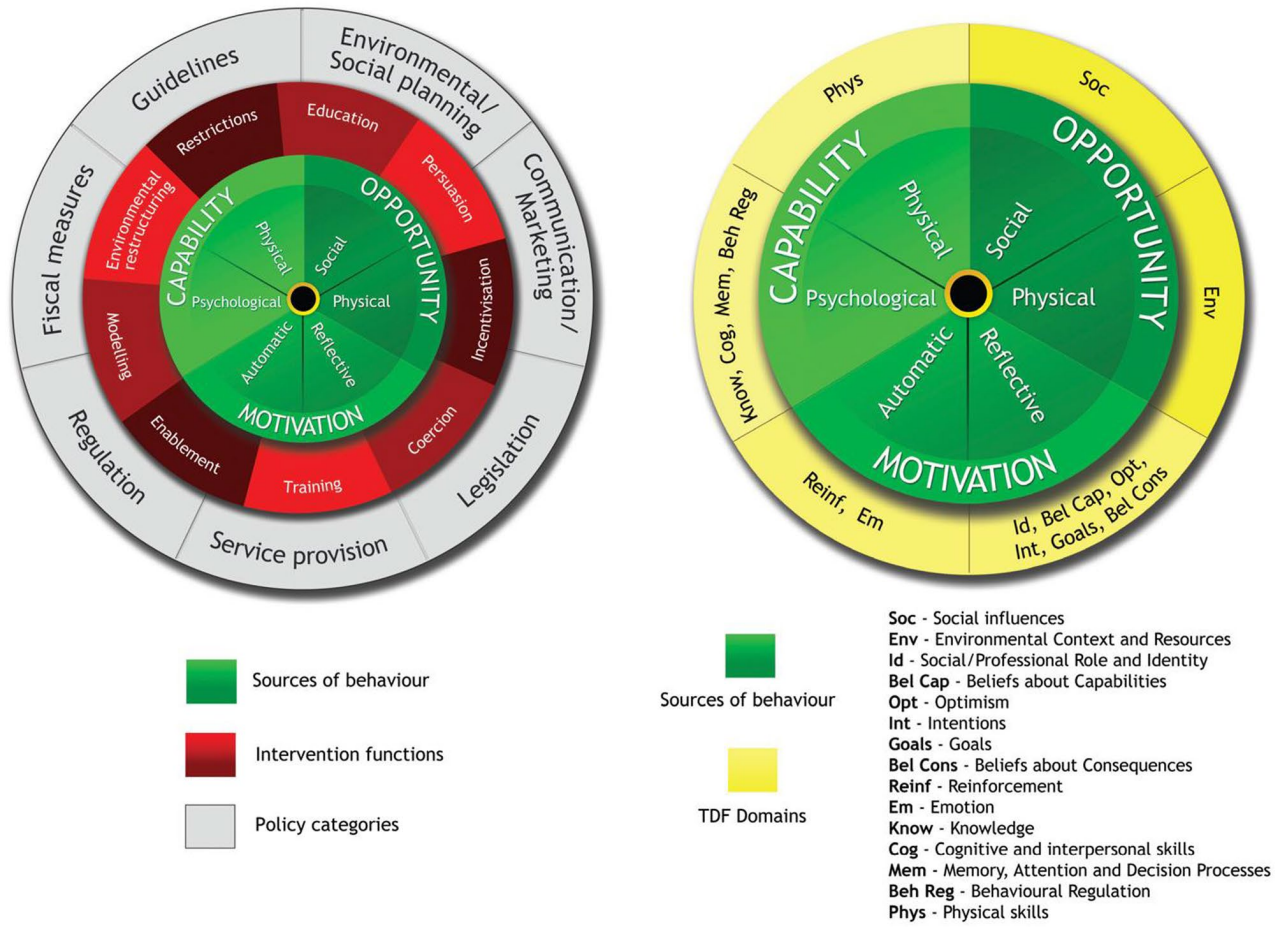
The Behaviour Change Wheel and the relationship with the Theoretical Domains Framework. Reprinted from [16]. Content may be subject to copyright

The use of a theoretical framework for structured identification and categorization of barriers to and enablers of implementation is highly recommended [7, 15]. In this study, the Behaviour Change Wheel (BCW) was used [4]. Supplementary to the COM-B model, the Theoretical Domains Framework (TDF) was used to separate the capability, opportunity, and motivation categories into 14 theoretical domains of behaviour (Fig. 1).

### Sampling strategy

ENT surgeons and ENT residents involved in providing care to patients with ear- or hearing-related complaints, received an email invitation from the research team to partake in the study. To ensure a representative sample of clinical hearing health care in the Netherlands, ENT surgeons and ENT residents from a secondary setting (NWZ and Deventer Hospital) and a tertiary setting (Amsterdam UMC) were included.

Patients were invited to participate in the study during the period from if they met the following inclusion criteria during the period from December 2022 to April 2023: (1) aged above 16 years, (2) visiting the ENT outpatient clinic with an ear or hearing complaint, (3) have been invited to complete the OQUA before their intake, pre- or post-operative consultation, (4) have an active digital account to access the OQUA in their own electronic patient record, (5) not familiar with a learning or cognitive disability, and (6) have a sufficient knowledge of the Dutch language. A maximum variation strategy was adopted in relation to age and ear- or hearing-related complaints to reflect heterogeneity within otology patient characteristics. There may be an overlap between the patients in the retrospective cohort (Table 1) and those who were invited for interview. Nevertheless, the patients of this qualitative post-implementation study are considered as a separate cohort, given the different time frame of the inclusions and the fact that patients who had not completed the OQUA or had completed it only once were also included.

### Data collection

Focus groups were organized for the ENT professionals and semi-structured interviews were conducted with patients of the Amsterdam UMC, NWZ and Deventer Hospital. The definitions, theoretical constructs and example interview questions in Supplementary Material S3 are based on the existing literature and were used to generate open ended and correctly phrased questions [15–18]. The interview and focus group schedule is provided in Supplementary Material S4. The topics of the focus groups and interviews were divided into four categories and were related to own experiences with the OQUA (Q1), enablers of using the OQUA (Q2), barriers to using the OQUA (Q3) and motivation to complete or use the OQUA during the consultation (Q4).

Each focus group consisted of 5 ENT professionals. The focus groups lasted one hour and were guided by an independent moderator. The moderator had no relationship with the ENT professionals and was unfamiliar with the use of the OQUA because of his non-medical background. One of the Authors [KV] was present as an observer and second interviewer to monitor and guide the process.

Interviews with patients were conducted by the researcher [KV]. The interview duration was 15–35 min. Data saturation was achieved when the study sample included the most common patient groups within otology practice and the last interview provided no new answers.

### Data analysis

The transcribed focus groups and interviews were imported to ATLAS.ti [19], which was used as a qualitative analysis software to efficiently edit and code the transcripts. The coding process was based on the content analysis technique of Atkins [15] and proceeded in four steps. First, the transcripts were classified into meaning units, considering their relevance to the definitions of the COM-B components and TDF domains. Second, the COM-B model was applied to subdivide the meaning units into the categories of capability, opportunity, and motivation. Third, these meaning units were further assigned based on the 14 theoretical domains of behaviour by using the TDF. Fourth, overarching themes were created to analyses and summarize the meaning units within the domains. The above coding process is illustrated using an excerpt from a transcript in Supplementary Material S5.

Three factors were considered to determine the key domains, which were previously applied in other published studies [15, 20]. First, the frequency of a theme was calculated by counting how often the theme was mentioned by one specific ENT professional or patient. Second, all themes were analysed independently for the presence of contrasting statements. Third, the perceived strength of a theme was calculated, which was based on how often the theme was mentioned as the biggest or most important barrier or enabler by one specific ENT professional or patient. A theme was considered relevant if it (1) was frequently mentioned as barrier or enabler, (2) did not contain conflicting statements and (3) was mentioned as an influential barrier of enabler of the performance of specific behaviour. Finally, the key domains were matched to their corresponding intervention functions and policy categories of the BCW (Supplementary Material S3). The Behaviour Change Techniques (BCT) [16] were used to provide suggestions for an implementation strategy.

## Results

A total of 15 ENT professionals and 25 patients participated in the study. Characteristics of the ENT professionals and patients are provided respectively in Tables 2 and 3.

**Table 2:** Characteristics of the ENT professionals (N = 15)

	Total	Amsterdam UMC	NWZ	Deventer Hospital
Number of participants	15	5	5	5
• ENT surgeons	14	5	5	4
• ENT residents	1	0	0	1
Gender *male/female*	10/5	5/0	2/3	3/2
Years of work experience *mean (range)*	13.2	15.8 (7–26)	13.4 (8–20)	10.3 (1–19)

**Table 3:** Characteristics of the patients (N = 25)

	Total	Amsterdam UMC	NWZ	Deventer Hospital
Number of participants	25	12	5	8
Gender *male/female*	13/12	7/5	3/2	3/5
Age (years) *mean (range)*	51.2 (25–81)	57.3 (30–81)	46.6 (31–67)	49.7 (25–75)
OQUA completed *yes/no*^a^	21/4	8/4	5/0	8/0
Otology diagnosis^a^				
*• Diseases of external ear*	2	2	0	0
*• Diseases of middle ear and mastoid*	20	7	5	8
*• Diseases of inner ear*	3	3	0	0

^**a**^Classification was based on the 10th revision of International Classification of Diseases and Related Health Problems

The identified factors are presented in tables based on the COM-B components and TDF domains, and are supported by themes. The perceived barriers to and enablers of the use of the OQUA are presented separately for ENT professionals in Tables 4, 5 and patients in Tables 6, 7. The bold domains are the most prominent barriers and enablers, based on frequency, presence of contrasting statements and perceived strength.

**Table 4:** Identified barriers to the use of the OQUA as perceived by ENT professionals

COM-B	TDF domain	Setting^a^	Theme^b^	FRQ	CS	PS
Psychological Capability	Knowledge	1,2,3	Lack of knowledge regarding the use of the OQUA in clinical practice	11/15	Yes	0/15
Physical Capability	Skills	1,2	Lack of skills to access the OQUA in the electronic patient record	8/15	Yes	0/15
Physical Capability	Skills	1,2,3	Lack of skills to interpret scores of the OQUA	7/15	Yes	0/15
**Reflective motivation**	**Beliefs about consequences**	1,2,3	Perceived lack of clarity of the clinical significance of the OQUA	**8/15**	**No**	**3/15**
**Physical Opportunity**	**Environmental context and resources**	1,2,3	Lack of IT support to (automate) use of the OQUA	**12/15**	**No**	**4/15**
Physical Opportunity	Environmental context and resources	1,2,3	Perceived (additional) time investment and administrative tasks to use the OQUA	8/15	Yes	5/15
Reflective motivation	Beliefs about consequences	1	Perceived obligation to review the results of the OQUA with the patient	3/15	No	0/15
Psychological capability	Behavioural regulation	1,2	Perceived resistance to change daily habits and routines	6/15	Yes	1/15
Reflective motivation	Optimism (pessimism)	2,3	Uncertainty whether the OQUA scores accurately reflect the severity of problems	4/15	Yes	0/15
Reflective motivation	Beliefs about consequences	2,3	Perceived fear to be judged by patient-defined quality	3/15	Yes	0/15
Automatic Motivation	Emotion	2,3	Perceived competition among hospitals	4/15	Yes	0/15
**Automatic Motivation**	**Reinforcement**	1,2,3	Lack of feedback regarding new insights based on the OQUA	**9/15**	**No**	**2/15**
Social opportunity	Social influences	1,2	Lack of triggers that encourage use of the OQUA	7/15	No	0/15
Physical opportunity	Environmental context and resources					

*Source*: *COM-B, TDF*, both adapted and reprinted from [16]. Content may be subject to copyright

^a^Setting: 1 = Amsterdam UMC, location VUmc, 2 = Noordwest Ziekenhuisgroep, location Alkmaar, 3 = Deventer Hospital

^b^Theme: represent a brief statement about the perceived barrier or enabler

*COM-B* Capability, Opportunity, Motivation – Behavior, *TDF* Theoretical Domains Framework, *FRQ* frequency of which a theme was mentioned among ENT professionals, *CS* presence of Contrasting Statements yes/no, *PS* Perceived Strength represents how often the theme was mentioned as the biggest or most important barrier

**Table 5:** Identified enablers of the use of the OQUA as perceived by ENT professionals

COM-B	TDF domain	Setting^a^	Theme^b^	FRQ	CS	PS
Physical opportunity	Environmental context and resources	1,2,3	Content-strong questionnaire to assess all relevant ear complaints and their severity in the clinical setting	9/15	No	0/15
**Reflective motivation**	**Beliefs about consequences**	1,2,3	Perceived value to measure effectiveness of interventions	**12/15**	**No**	**5/15**
**Reflective motivation**	**Goals**	1,2,3	Assigned importance of learning from the outcomes as an individual, department and/or organization	**8/15**	**No**	**2/15**
**Reflective motivation**	**Goals**	1,2,3	Assigned relevance of being able to compare and share outcomes in research settings	**8/15**	**No**	**3/15**
Reflective motivation	Social/professional role and identity	2,3	Experienced professional obligation of being able to socially justify your medical interventions	4/15	No	0/15
Reflective motivation	Social/professional role and identity	1,3	Experienced scientific obligation to maintain classification as a top clinical or academic hospital	4/15	No	0/15
**Reflective motivation**	**Beliefs about consequences**	1,2,3	Perceived value to identify, capture and quantify all of the patient’s relevant ear complaints	**11/15**	**No**	**3/15**
Reflective motivation	Goals	1,2,3	Assigned importance to proper preparation for consultation or anamnesis	7/15	No	0/15
**Reflective motivation**	**Goals**	1,2,3	Awareness of discrepancy between doctors’ and patients’ perceptions	**8/15**	**No**	**2/15**
Reflective motivation	Social/professional role and identity	1,2	Experienced professional obligation to assess patients’ relevant complaints	3/15	No	0/15

*Source*: *COM-B, TDF,* both adapted and reprinted from [16]. Content may be subject to copyright

^**a**^Setting: 1 = Amsterdam UMC, location VUmc, 2 = Noordwest Ziekenhuisgroep, location Alkmaar, 3 = Deventer Hospital

^**b**^Theme: represent a brief statement about the perceived barrier or enabler

*COM-B* Capability, Opportunity, Motivation – Behavior, *TDF* Theoretical Domains Framework, *FRQ* frequency of which a theme was mentioned among ENT professionals, *CS* presence of Contrasting Statements yes/no, *PS* Perceived Strength represents how often the theme was mentioned as the biggest or most important barrier

**Table 6:** Identified barriers to the use of the OQUA as perceived by patients

COM-B	TDF domain	Setting^a^	Theme^b^	FRQ	CS	PS
**Psychological Capability**	**Knowledge**	1,2,3	Lack of knowledge about the objective, content and usefulness of the OQUA	**19/25**	**Yes**	**3/25**
Psychological Capability	Memory, attention and decision processes	1,2,3	Experienced challenge in recognizing the importance of the OQUA among large supply of questionnaires	10/25	Yes	1/25
**Reflective Motivation**	**Beliefs about consequences**	1,2,3	Lack of insight about the relevance of completing the OQUA to the patient as an individual	**15/25**	**No**	**4/25**
Physical Opportunity	Environmental context and resources	1,2	Lack of clarity about the questions and structure of the OQUA	6/25	Yes	0/25
Physical Capability	Skills	1,2	Lack of skills to interpret the questions of the OQUA, and to report the complaints correctly	6/25	Yes	3/25
**Physical Opportunity**	**Environmental context and resources**	1,3	Lack of opportunity to add personal comments to the OQUA	**8/25**	**No**	**4/25**
Physical Opportunity	Environmental context and resources	2,3	Experienced time investment in order to complete the OQUA	6/25	No	3/25
Physical Opportunity	Environmental context and resources	1,2	Lack of opportunity to complete the OQUA at the outpatient clinic	6/25	Yes	0/25
Reflective motivation	Beliefs about consequences	1,2	Lack of linkage between OQUA and a consultation appointment	4/25	Yes	0/25
**Automatic Motivation**	**Reinforcement**	1,2,3	Insufficient feedback on the results of the OQUA in the consultation	**14/25**	**No**	**7/25**
Automatic Motivation	Reinforcement	1	Lack of (graphical) overview of scores in the electronic patient record after completing the OQUA	2/25	No	0/25

*Source*: *COM-B, TDF,* both adapted and reprinted from [16]. Content may be subject to copyright

^**a**^Setting: 1 = Amsterdam UMC, location VUmc, 2 = Noordwest Ziekenhuisgroep, location Alkmaar, 3 = Deventer Hospital

^**b**^Theme: represent a brief statement about the perceived barrier or enabler

*COM-B* Capability, Opportunity, Motivation – Behavior, *TDF* Theoretical Domains Framework, *FRQ* frequency of which a theme was mentioned among ENT professionals, *CS* presence of Contrasting Statements yes/no, *PS* Perceived Strength represents how often the theme was mentioned as the biggest or most important barrier

**Table 7:** Identified enablers of the use of the OQUA as perceived by patients

COM-B	TDF domain	Setting^a^	Theme^b^	FRQ	CS	PS
Physical Opportunity	Environmental context and resources	1,2,3	Experienced adequate support to access the OQUA	18/25	Yes	0/25
Physical Opportunity	Environmental context and resources	1,2,3	Content-strong questionnaire that covers all relevant ear complaints	8/25	No	0/25
Reflective motivation	Beliefs about consequences	1,3	Perceived value of increased time efficiency during consultation	3/25	No	1/25
**Reflective motivation**	**Beliefs about consequences**	1,2,3	Perceived value of better preparation for consultation and improved understanding of own complaints	**13/25**	**No**	**4/25**
Reflective motivation	Goals	1	The willingness to participate in decision-making about the treatment or intervention	3/25	No	0/25
Reflective motivation	Goals	1,3	The need of being able to discuss the impact of complaints	6/25	No	0/25
**Reflective motivation**	**Beliefs about consequences**	1,2,3	Perceived value of providing feedback to support other patients, the organization or science	**21/25**	**No**	**15/25**
**Reflective motivation**	**Goals**	1,2,3	The desire to contribute to the improvement of health care	**17/25**	**No**	**5/25**

*Source*: *COM-B, TDF*, both adapted and reprinted from [16]. Content may be subject to copyright

^**a**^Setting: 1 = Amsterdam UMC, location VUmc, 2 = Noordwest Ziekenhuisgroep, location Alkmaar, 3 = Deventer Hospital

^**a**^Theme: represent a brief statement about the perceived barrier or enabler

*COM-B* Capability, Opportunity, Motivation – Behavior, *TDF* Theoretical Domains Framework, *FRQ* frequency of which a theme was mentioned among ENT professionals, *CS* presence of Contrasting Statements yes/no, *PS* Perceived Strength represents how often the theme was mentioned as the biggest or most important barrier

### Key barriers and enablers perceived by ENT professionals

Table 4 shows all identified barriers to the use of the OQUA as perceived by ENT professionals. The three key barriers are underlined in Table 4. The key barriers were reinforced by the observation that more than one third of the ENT professionals indicated that they had to get accustomed of using PROMs in consultation, meaning that analysing and discussing outcomes of the questionnaire is not (yet) embedded as a standard part of their daily routines or system. The reported belief that a professional does not need a PROM to figure out a patient’s main complaint and question, and the quote below illuminate this further.

In my daily practice, the use of PROMs is not (yet) embedded, it’s just not in my system. You have been doing the profession for a long time, even without OQUA. Then I think, I practiced the profession well in that time, didn’t I?

Table 5 shows all identified enablers of the use of the OQUA as perceived by ENT professionals. Five key enablers are highlighted in Table 5. These enablers are supported by the quote below.

The outcomes are illuminating to me. I’m getting the feedback and that’s is instructive; You’ll see, I closed 10 eardrums and all patients are now suffering from pressure on the ear. It’s clear to me from these answers that I have to change my treatment strategy. We have to learn from our results.

### Key barriers and enablers perceived by patients

In Table 6 all barriers as perceived by patients in the use of the OQUA are stated. Four key barriers are highlighted in Table 6. In particular, more than half of the patients reported insufficient feedback on the results of the OQUA in the consultation as a major barrier. They indicated that their motivation could be increased if the outcomes of the OQUA had been clearly named and discussed by the ENT-professional. In the current situation, it was not transparent what results could be achieved with the OQUA and when these results would be evaluated with the patient.

The main incentive to complete the questionnaire is if I know the doctor is going to do something with it. That he gives feedback: I have seen your answers, and based on that, I’m going to do this … Then it becomes clear, completing the questionnaire is important for you and me.

Table 7 shows all identified enablers of the use of the OQUA as perceived by patients. Three key enablers are highlighted in Table 7. In general, the patient’s motivation to complete the OQUA was high as the results can be used to provide the ENT professional, department or organization with information and feedback about the treatment. Patients perceived the OQUA as an appropriate method of expressing clearly what they experience, what they encounter or what they still need. In particular, the ability to share these results and feedback was considered by most patients as the main incentive to complete the OQUA, as it can improve the care for other patients and broaden the science. The following quote illustrates these findings.

Through the results of the questionnaire, you get more insight into the patient, in terms of: what does the patient think, what does the patient feel, what does the patient want? That’s what makes health care better. For another person, but also for me.

### Implementation strategy

The key barriers and enablers were mapped to their corresponding intervention functions and policy categories of the BCW (Supplementary Material S3) and can be found in Tables 8 and 9. The BCT were used as examples for determining the content of the interventions. Implementation strategy should focus on education and training about the objective, outcomes and relevance of the OQUA, environmental restructuring regarding the optimal use of the OQUA, and incentivisation with feedback on the valuable outcomes of the OQUA for the patient, professional and healthcare.

**Table 8:** Implementation strategy based on the identified key barriers

COM-B	TDF	Key barriers^a^	Intervention^b^	Policy^b^	Strategy suggestion
**Capability** Psychological capability	Knowledge	Lack of knowledge regarding the use of the OQUA (E)	Education	Communication/marketing, Service Provision	• Organizing online/physical meetings with ENT professionals to provide information on the content, objective and outcomes of the OQUA: when to use, how to use, why to use? • Making the above information available in a (digital) document for reference in clinical practice.
		Lack of knowledge about the objective, content and usefulness of the OQUA (P)	Education	Communication/marketing, Service provision	• Developing supporting text as an introduction to the OQUA in the electronic patient record to provide information on content, objective and outcomes of the OQUA: why to complete, how to complete, what (individual) benefits to expect? • Making the above information available in a flyer to give to the patient after their consultation at outpatient clinic.
**Capability** Physical capability	Skills	Lack of skills to access the OQUA (E)	Training	Communication/marketing, Service Provision	• Organizing online/physical meetings with ENT professionals to demonstrate the access and use of the OQUA in the electronic health record: how to use, how to interpret, how to document? • Making the above information available in an instruction video for reference in clinical practice
		Lack of skills to interpret the scores (E)	Training	Communication/marketing, Service Provision, Guidelines	• Developing guidelines to interpret OQUA scores: offering normal values and the minimal important change (both currently in progress) • Offering clinical scenarios during the meeting that allow practice with the guidelines, followed by (individual) feedback
**Opportunity** Physical opportunity	Environmental context and resources	Lack of IT support to (automate) use the OQUA (E)	Environmental restructuring	Environmental planning	• Engaging IT technicians to enhance visibility and accessibility of the OQUA in electronic health record • Evaluation of the OQUA pathway with IT expert team to optimize automatisation (order request to processing results) • Facilitating a smartphrase and/or graphical overview to present results directly in the anamnesis format • Providing (computerized) reminders of the use in key clinical area
		Lack of opportunity to add personal comments to the OQUA (P)	Environmental restructuring	Environmental planning	• Evaluating and discussing with IT expert team and ENT professionals the opportunity of allowing the patient to give a brief explanation of the answers at the end of the OQUA • See strategy example of knowledge (education about the content and structure of the OQUA and how to complete the OQUA at home or by mobile) • Providing an example to illustrate the (previously difficult reported) questions
**Motivation** Reflective motivation	Beliefs about consequences	Unclarity of the clinical significance of the OQUA (E)	Education (including aspects of persuasion and modelling)	Communication/marketing, Guidelines	• See strategy example of knowledge (education about the outcomes of the OQUA), skills (training for interpreting scores, support by guidelines) and reinforcement. (feedback on achieved results in the clinical practice) • Demonstrating clinical scenarios in which the OQUA can be (successfully) used during consultation, to determine policies and/or interventions with the patient.
		Lack of insight about the relevance and outcomes of the OQUA (P)	Education (including aspects of persuasion and modelling)	Communication/marketing, Service provision	• See strategy example of knowledge (education about the outcomes and individual benefits of the OQUA) and reinforcement (providing and explaining the individual results of the OQUA to the patient) • Illustrating the above with (graphic) examples and/or quotations from other patients about experienced relevant outcomes in the flyer or supporting text of the OQUA
**Motivation** Automatic motivation	Reinforcement	Lack of feedback regarding new insights based on the OQUA (E)	Incentivisation	Communication Service provision Guidelines	• Recording all pre- and post-operative scores since implementation of the OQUA (currently completed) • Providing an overview of the above results: comparing pre- and postoperative scores, categorized by diagnosis and/or intervention (currently in progress, data collection and -analysis ongoing). • Organizing online/physical meeting with the ENT professionals to explain and evaluate results, as for determining deployment of the OQUA in research settings. • Repeating the above every six months to year, if necessary in combination with education and training meetings.
		Insufficient feedback on the results of the OQUA in consultation (P)	Incentivisation Environmental restructuring	Communication Service provision	• Providing an overview of individual scores to the patient during consultation and/or by offering graphical representation in patient’s personal electronic record • Offering explanation to the patient about the expected results after post-operative measurements (following strategy example of knowledge)

^a^*E* ENT professionals, *P* Patients

^b^Intervention functions and policies are reprinted from [16]. Content may be subject to copyright

**Table 9:** Implementation strategy based on the identified key enablers

COM-B	TDF	Key enabler^a^	Intervention^b^	Policy^b^	Strategy suggestion
**Motivation** Reflective motivation	Beliefs about consequences	Perceived value to measure effectiveness of interventions (E)	Education (including aspects of persuasion and modelling)	Communication/marketing, Service provision, Guidelines	• See strategy suggestion of reinforcement (providing an overview with the differences between pre- and postoperative scores, evaluating these results in meeting) • Highlighting examples of the results to discuss the current practices in the meeting (see goals)
		Perceived value to identify, capture and quantify all of the patient’s relevant ear complaints (E)	Education (including aspects of persuasion and modelling)	Communication/marketing, Service provision, Guidelines	• See strategy example of knowledge (education about the outcomes of the OQUA), skills (training for interpreting scores, support by guidelines) and beliefs about consequences (demonstrating clinical scenarios in which the OQUA can be (successfully) used during consultation)
		Perceived value of better preparation for consultation and improved understanding of own complaints (P)	Education (including aspects of persuasion and modelling)	Communication/marketing, Service provision	• See strategy suggestion of knowledge (developing supporting text about the content, objective and outcomes of the OQUA) • Mentioning individual benefits in the supporting text: e.g. better preparation of the interview, more insight into own complaints, enhanced participation in shared-decision-making and increased time efficiency during the consultation. • Making the above information available in a flyer to give to the patient after their consultation at outpatient clinic.
		Perceived value of providing feedback to support other patients, the organization or science (P)	Education (including aspects of persuasion and modelling)	Communication/marketing, Service provision	• See above • Mentioning the general benefits for health care in the supporting text: e.g. evaluating all pre- and postoperative scores will help in comparing and improving treatments from the patients’ perspective
**Motivation** Reflective motivation	Goals	Assigned importance of learning from the outcomes as an individual, department and/or organization (E)	Education (including aspects of persuasion and modelling) Incentivisation	Communication/marketing, Service provision	• See strategy suggestion of reinforcement (providing an overview with the differences between pre- and postoperative scores) and beliefs about consequences (discussing the current clinical practices based on the results) • Evaluating differences between individuals, departments of organizations • Organizing sessions to observe the work of others
		Assigned relevance of being able to compare and share outcomes in research settings (E)	Education (including aspects of persuasion and modelling) Incentivisation	Communication/marketing, Service provision	• See strategy suggestion of reinforcement (providing an overview with the differences between pre- and postoperative scores) and beliefs about consequences (evaluating and discussing the current clinical practices based on the results) • Considering which treatment options need to be evaluated with the OQUA (surgical and non-surgical) • Setting up research settings and providing this feedback according to the strategy of reinforcement
		Awareness of discrepancy between doctors’ and patients’ perceptions (E)	Education (including aspects of persuasion and modelling) Incentivisation	Communication/marketing, Service provision	• See strategy suggestion of reinforcement (providing an overview with the differences between pre- and postoperative scores) and beliefs about consequences (evaluating and discussing the current clinical practices based on the results) • Highlighting examples where the objective outcome (e.g. audiogram) does not match the subjective outcomes (pre- and postoperative scores OQUA)
		The desire to contribute to the improvement of health care (P)	Education (including aspects of persuasion and modelling)	Communication/marketing, Service provision	• See strategy suggestion of knowledge (developing supporting text about the content, objective and outcomes of the OQUA) and beliefs about consequences (mentioning the general benefits for health care)

^a^*E* ENT professionals, *P* Patients

^b^Intervention functions and policies are reprinted from [16]. Content may be subject to copyright

## Discussion

The application of the implementation strategy approach may contribute to enhancing the uptake of the OQUA in clinical practice. This study provides a foundation for developing interventions aimed at overcoming barriers and/or strengthening enablers, in order to realize the potential benefits of the OQUA on patient-centred care within otology practice.

The findings in our study are consistent with the most reported barriers in the literature [3, 6, 12, 13], which supports our hypothesis. Several studies reported the insufficient knowledge of individual clinicians about PROMs as an influential barrier [6, 12, 13], as it can affect their ability to interpret scores and their confidence in skills to use PROMS in clinical decisions [21–23]. In addition, it is recognized that if PROMS are not considered useful in the clinical decision-making process, this may further limit their implementation [13]. In the case of the OQUA, ENT professionals received very little information before, during or after implementation about the content, objectives and outcomes. In addition, no information was available as a reference guide to facilitate the use and interpretation of the OQUA.

Previous studies have captured the technological barriers of PROMs, which mainly referred to the limited integration, accessibility and visibility of PROMs in the electronic patient record [3, 9, 24]. These barriers are in line with the reported problems in our study and can be explained by the lack of availability of user-friendly and reliable software and hardware for PROM integration [12]. The technological limitations appear to have a significant impact on the effectiveness of the uptake of PROMs into clinical practice [25].

Studies have suggested that the skepticism about the usefulness may be caused by the professionals’ lack of awareness and understanding of the value of PROMs for clinical practice [13, 26]. The underestimated value of the OQUA may be explained by the lack of received information and feedback about the outcomes, completion of training before PROMs were part of the curriculum (average of 13.2 years of work experience) and, in addition, general resistance to change in an established workflow [3, 12].

In the literature, the overwhelming amount of patient information provided by a PROM is considered a potential barrier to its use in clinical practice [12, 21, 23]. This barrier was not identified in our study, which may be explained by the intensive development process of the OQUA with involvement and feedback from ENT professionals, and by the reported belief that the OQUA covers all relevant ear complaints. However, this (potential) barrier should be included in the careful consideration of allowing patients to explain their answers in the questionnaire.

Enablers have been shown to be more variable across health care settings than barriers [3]. The variation observed in the literature may be explained by differences in the implementation stage (pre-, during or post), the local needs of clinics, specific healthcare settings and different patient populations. These enablers included the convenience of tracking and monitoring patient changes, allowing analysis among patients, colleagues and organization; validating a professional’s clinical perception of patient outcomes; facilitating the extraction of patient information that may not be discussed during the consultation; and strengthening the patient-clinician relationship [6, 12]. The above factors can be explained by their consistency with the objectives of the OQUA and in general by the potential benefits of PROMs on providing patient-centred care [1, 2]. This concordance may underscore the importance of educating professionals about the objectives of the OQUA, demonstrating the beneficial outcomes of the OQUA as a PROM by training, and evaluating the results of the OQUA with individuals, departments or organizations for incentivisation.

Looking from the patients perspective, often the majority of patients are not familiar with the content, purpose and benefits of PROMs in general [12, 27]. This observation can be supported with the findings of our study. One explanation for this barrier may be that most patients (compared to healthcare providers) do not encounter PROMs in their daily lives, and in case of the OQUA, patients did not receive insufficient information from the professional or organization. Several studies have suggested that providing educational resources (e.g., brochures, videos) or offering support from staff can be helpful in increasing patients’ general knowledge about PROMs [12, 28].

In the literature, the perceived burden of completing PROMs is described as one of the biggest barriers for the patient. This implies that a PROM should be short (recommendation of a maximum time investment of 15 min), user-friendly, and in addition, clinically relevant [5, 12]. Based on the findings of our study, patients were willing to invest this time but lacked an understanding of the clinical impact of the OQUA. This can be explained by the missing link between OQUA and the patient’s appointment and the lack of feedback on the results of the OQUA during the consultation. In addition, previous research has shown that the compliance of PROMs can be increased when the patient knows that their answers are used by the professional in the consultation [29]. Although this supports our implementation strategy of incentivisation, ENT professionals have also reported a barrier in the requirement to provide patients feedback on the results of the OQUA. It is important to take this contradiction into account during the development of the implementation strategy. Nevertheless, providing overview of individual outcomes to the patient seems necessary for increasing clinical impact and lowering the perceived burden of the OQUA.

Studies have frequently recorded patients’ perceived difficulties in completing PROMs. These problems were mainly related to PROMs that were too difficult, too confusing or too anxiety-provoking and seemed to be reported primarily by patients with physical or cognitive impairments, or limited computer and language skills [12, 13]. In our study, the generalizability and interpretability of the questions were discussed as potential difficulties in the use of the OQUA. However, this was only reported as a barrier by a small proportion of patients. On the one hand, this can be explained by previous validation of the OQUA among a large group of patients [11]. On the other hand, it is possible that this barrier was underreported in our study due to of the exclusion of patients with a learning disability or cognitive impairment, or insufficient understanding of the Dutch language, the underrepresentation of patients who did not complete the OQUA, and because of the additional information and guidance provided to patients of Deventer Hospital during completion of the OQUA. Furthermore, the educational level of the patients was not taken into account. This may have had an effect of (under)reporting barriers in a patient’s ability to find, understand and use the OQUA, known as the concept of ‘health literacy’ [30]. These observations, combined with patients’ reported desire to explain their answers, suggest that further research may be needed to draw definitive conclusions about the comprehensibility of the OQUA.

The patients’ identified enablers were consistent with the facilitating factors observed in the literature. It is considered to be important to emphasize the individual and generic benefits of the OQUA in supporting information and education to patients.

### Strength and weakness of the study

The assurance of trustworthiness is an important construct in conducting qualitative research, which can be compared to validity and reliability in quantitative research. The strengths of this study are related to the three components of trustworthiness: credibility, transferability and dependability [31].

First, we feel that we ensured credibility in this study by using the TDF and COM-B model as part of the BCW, whose suitability for PROM implementation has been previously demonstrated [3, 5]. Moreover, we aimed to enhance the credibility of this study by using the concept of triangulation through the process. This is evidenced by the inclusion of both user groups of the OQUA (ENT professionals and patients of one tertiary referral hospital and two regional hospitals), the consideration of the characteristics of both groups in choosing data collection methodologies and the involvement of two researchers and one independent moderator in the data collection and analysis. Second, we attempted to ensure the transferability of this study by providing a representative quotes for the identified barriers and enablers. We believe that this may provide sufficient detail for the reader to assess whether the results of this study can be related to similar contexts. Third, in order to ensure the dependability of this study, we used the four-phase content analysis technique of Atkins [15]. We aimed to capture the analysis process as accurately as possible, by documenting all research steps and providing the used supporting information.

Data saturation was not reached in the group of ENT-professionals. Due to the limited availability of professionals with a specialization in otology, we were unable to organize more focus groups. Therefore, some barriers and enablers may have been missed. However, there was a large overlap in identified factors among the focus groups, which suggests that the lack of data saturation may be restricted. Due to logistical and organizational restrictions, it was not possible to reflect the heterogeneity within characteristics of otology patients from NWZ and Deventer hospital. As a result, patient groups with external or inner ear diseases and patients who did not complete the OQUA were underrepresented. This may explain the previously achieved theoretical saturation at both hospitals. This implies that some barriers or factors may have been overlooked, or that the effect of the identified factor may not be adequately recognized.

Applying frequency counts in a qualitative study can be debated, given there was no representative sample size. However, the determination of key domains was necessary for developing an implementation strategy, considering the large amount of identified barriers and enablers in our study. We attempted to overcome this limitation by incorporating recommendations from previous implementation studies [15, 20].

The OQUA was used in research settings at Deventer Hospital, meaning that patients were guided and supported in completing the questionnaire. This may have affected the non-reporting of capability- and opportunity barriers, possibly biasing the identification of motivation barriers. Nevertheless, this does not directly impact the suggestions of the implementation strategy, given that this strategy focuses on addressing barriers from all categories of the COM-B model. In addition, the findings from Deventer Hospital may be useful for developing (successful) interventions at Amsterdam UMC and NWZ to overcome their capability- and opportunity barriers.

Due to logistical and organizational constraints, we have presented only the perspective of OQUA users. Barriers and enablers may have been disregarded by not demonstrating the perspective of other stakeholders. In particular, the perspective of nurses and other professionals working in auditory rehabilitation, given their direct involvement and guidance of patients before, during and after the surgical intervention. It is plausible that this group therefore has a close understanding of what patients experience, need and would like to change in the use of PROMs in clinical practice. The inclusion of their perspective may have contributed to developing and prioritizing the interventions of the implementation strategy. Other examples include outpatient assistants, ICT technicians or non-nursing staff involved in the organization. Including their perspective would have been valuable, as it may influence the feasibility of the implementation strategy.

### Implications of the study

The results of this study are needed for the final development of an implementation strategy in order to improve the clinical usefulness of the OQUA. Additional research may focus on the elaboration of this implementation strategy by assessing the proposed interventions in terms of Affordability, Practicality, (cost-) Effectiveness, Acceptability, Safety and Equity (APEASE criteria by Michie, Atkins [16]. In addition, we recommend the use of current evidence on interventions from other implementation studies [3, 12, 13] and the involvement of relevant stakeholders in the final selection and deployment of interventions. While executing the implementation strategy, future research may focus on re-evaluating the clinical use of the OQUA. Indeed, we anticipate that the implementation of the OQUA will be an ongoing process, which requires continuous evaluations and modifications.

In general, we expect that the findings of this study will be informative for healthcare providers and for applying the IS approach to improve PROM implementation. Because of the large overlap between the identified barriers and enablers in our study and those in the literature, we suggest that incorporating these factors into (pre-)implementation processes may contribute to the successful uptake of PROMs in clinical practice.

## Conclusion

This study has demonstrated that barriers to the use of the OQUA are related to the capability, opportunity and motivation of ENT professionals and patients. The identified enablers have shown that both groups are motivated to incorporate the OQUA into their daily practices. These findings highlight that interventions are needed to improve implementation of the OQUA. We suggest that the implementation strategy should focus on education and training about the objective, outcomes and relevance of the OQUA, environmental restructuring regarding the optimal use of the OQUA, and incentivisation with feedback on the valuable outcomes of the OQUA for the patient, professional and healthcare.

### Electronic supplementary material

Below is the link to the electronic supplementary material.

Supplementary Material 1

Supplementary Material 2

Supplementary Material 3

Supplementary Material 4

Supplementary Material 5

## Data Availability

The datasets used and/or analysed during the current study are available from the corresponding author on reasonable request.
