# Supplementary material for: Barriers to and enablers of the use of the Otology Questionnaire Amsterdam in clinical practice—a qualitative post-implementation study
Source: J Patient Rep Outcomes. 2024 Aug 14;8:96. doi: 10.1186/s41687-024-00741-9 (PMC11324631; doi:10.1186/s41687-024-00741-9)
Supplement: Supplementary file 1 — Supplementary Material 1 [file 41687_2024_741_MOESM1_ESM.docx]

**S1. Validated version of the ‘Otology Questionnaire Amsterdam’**

Reprinted from “The Otology Questionnaire Amsterdam: A generic patient-reported outcome measure about the severity and impact of ear complaints. Validation, reliability and responsiveness” by Kraak, J. T., van Dam, T. F., van Leeuwen, L. M., Kramer, S. E., & Merkus, P. (2020). Clin Otolaryngol, 45(4), 506-516. https://doi.org/10.1111/coa.13545

# Complaints:

|  | (Almost)  never | Sometimes | Regularly | Often | (Almost)  always |
| --- | --- | --- | --- | --- | --- |
| 1 | I have an earache |  |  |  |  |

2 Indicate the severity of your earache on the line below.


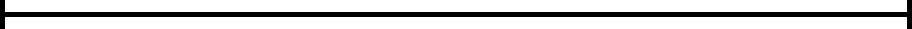


|  | (Almost)  never | Sometimes | Regularly | Often | (Almost)  always |
| --- | --- | --- | --- | --- | --- |
| 3 | I feel pressure in my ear. |  |  |  |  |
| 4 | My ear pops. |  |  |  |  |

5. Indicate the severity of pressure in your ear on the line below.


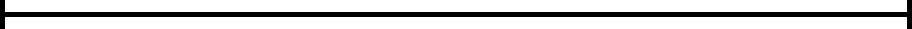


|  | (Almost)  never | Sometimes | Regularly | Often | (Almost)  always |
| --- | --- | --- | --- | --- | --- |
| 6. | I have an itch in or on my ear. |  |  |  |  |

7. Indicate the severity of itching in or on your ear to the line below.


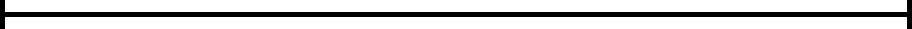


|  | (Almost)  never | Sometimes | Regularly | Often | (Almost)  always |
| --- | --- | --- | --- | --- | --- |
| 8. | I hear a hum, murmur, beeping noise or buzzing  sound. |  |  |  |  |

9. Indicate the severity of your tinnitus (this can be a hum, murmur, beeping noise or buzzing sound) on the line below.


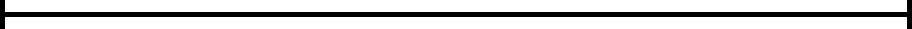


|  | (Almost)  never | Sometimes | Regularly | Often | (Almost)  always |
| --- | --- | --- | --- | --- | --- |
| 10 | Can you hear somebody approaching from  behind? |  |  |  |  |
| 11 | Can you hear cars passing by? |  |  |  |  |
| 12 | Can you hear from what corner of a room someone is talking to you being in a quiet  house? |  |  |  |  |
| 13 | Can you understand the presenter of the news on  TV at a normal volume? |  |  |  |  |
| 14 | Can you follow a conversation between a few people during dinner? |  |  |  |  |
| 15 | I am sensitive to loud noises. |  |  |  |  |

16. Indicate the severity of your hearing loss on the line below.


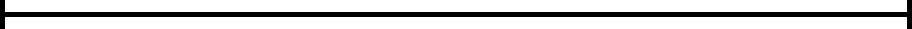


|  | (Almost)  never | Sometimes | Regularly | Often | (Almost)  always |
| --- | --- | --- | --- | --- | --- |
| 17 | Liquid comes out of my ear. |  |  |  |  |
| 18 | Pus comes out of my ear. |  |  |  |  |

19. Indicate the severity of your ear discharge on the line below.


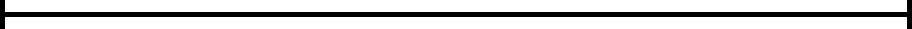


|  | (Almost)  never | Sometimes | Regularly | Often | (Almost)  always |
| --- | --- | --- | --- | --- | --- |
| 20 | I have a poor sense of taste |  |  |  |  |

21. Indicate the severity of your loss of taste on the line below.


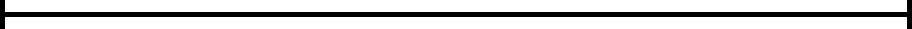


|  | (Almost)  never | Sometimes | Regularly | Often | (Almost)  always |
| --- | --- | --- | --- | --- | --- |
| 22 | I have balance problems. |  |  |  |  |
| 23 | I feel dizzy |  |  |  |  |
| 24 | When I move my head I get dizzy. |  |  |  |  |

25. Indicate the severity of your dizziness on the line below.


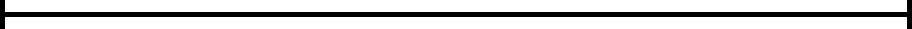


# Impact:

|  | Strongly  disagree | Disagree | Neutral | Agree | Strongly  agree |
| --- | --- | --- | --- | --- | --- |
| 26 | I get irritated due to my ear problems. |  |  |  |  |
| 27 | I get upset due to my ear problems. |  |  |  |  |
| 28 | I have impaired concentration due to my ear  problems. |  |  |  |  |
| 29 | I feel depressed due to my ear problems. |  |  |  |  |
| 30 | My ear problems are very tiring. |  |  |  |  |
| 31 | My ability to take part in social activities (hobbies, sport or leisure-time activities) is  limited due to my ear problems. |  |  |  |  |
| 32 | I have had to modify my daily activities and/or  work due to my ear problems. |  |  |  |  |
| 33 | My ear problems make life difficult for me. |  |  |  |  |
| 34 | I am concerned about my ear problems. |  |  |  |  |

**Figure 1:** OQUA questionnaire (English translation, Original language Dutch)

The questionnaire contains 34 items of which 9 are about Impact and 25 about the 8 most prominent ear complaints (Earache, Pressure sensation, Itching, Tinnitus, Hearing loss, Ear discharge, Loss of taste, Dizziness). Each complaint has one question in VAS scale to score the severity of the complaint. 18 questions with a 5- likert scale answer category.
