# Supplementary figures and images for: Barriers to and enablers of the use of the Otology Questionnaire Amsterdam in clinical practice—a qualitative post-implementation study
Source: J Patient Rep Outcomes. 2024 Aug 14;8:96. doi: 10.1186/s41687-024-00741-9 (PMC11324631; doi:10.1186/s41687-024-00741-9)

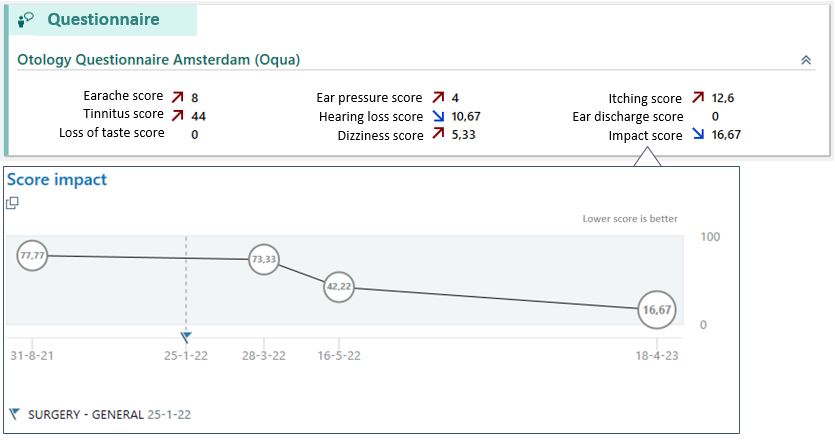

Supplement: Supplementary file 2 — Supplementary Material 2 [file 41687_2024_741_MOESM2_ESM.jpg]
