# Supplementary material for: Barriers to and enablers of the use of the Otology Questionnaire Amsterdam in clinical practice—a qualitative post-implementation study
Source: J Patient Rep Outcomes. 2024 Aug 14;8:96. doi: 10.1186/s41687-024-00741-9 (PMC11324631; doi:10.1186/s41687-024-00741-9)
Supplement: Supplementary file 3 — Supplementary Material 3 [file 41687_2024_741_MOESM3_ESM.docx]

S3. COM-B components linked to the related TDF domains and to the BCW intervention functions and policy categories.

Reprinted and adapted from “The Behaviour Change Wheel: A guide to designing interventions” (p. 63, 88-90, 111-115, 136-137) by Michie, S., Atkins, L., West, R., 2014, UK: Silverback Publishing.

| **COM-B component** *definition* | **TDF domain**  *definition* | **Intervention function**  *definition* | **Policy categories** |
| --- | --- | --- | --- |
| **Capability**  **Physical Capability**  Physical skill, strength or stamina | **Skills**  An ability or proficiency acquired through practice | **Training**  Imparting skills | Guidelines  Fiscal measures  Regulation Legislation  Service provision |
| **Capability**  **Psychological capability**  Knowledge or psychological skills, strength or stamina to engage in the necessary mental processes | **Knowledge**  An awareness of the existence of something | **Education**  Increasing knowledge or understanding | Communication/marketing Guidelines  Regulation  Legislation  Service provision |
| **Capability**  **Psychological capability** | **Memory, attention and**  **decision Processes**  The ability to retain information, focus  selectively on aspects  of the environment and  choose between two or  more alternatives | **Training**  Education  **Environmental restructuring**  Changing the physical or social context  **Enablement**  Increasing means/reducing barriers to increase capability (beyond education and training) or opportunity (beyond environmental restructuring) | Guidelines  Communication/marketing  Fiscal measures  Regulation  Legislation  Environmental/social planning  Service provision |
| **Capability**  **Psychological capability** | **Behavioural regulation**  Anything aimed at  managing or changing  objectively observed or  measured actions | **Training**   **Education**  **Enablement**  **Modelling**  Providing an example for people to aspire to or imitate | Guidelines  Communication/marketing  Fiscal measures  Regulation Legislation Environmental/social planning  Service provision |
| **Opportunity**  **Physical opportunity**  Opportunity afforded by the environment involving time, resources, locations, cues, physical ‘affordance’ | **Environmental context**  **and resources**  Any circumstance of  a person’s situation  or environment that  discourages or encourages  the development of  skills and abilities,  independence, social  competence, and adaptive  behaviour | **Training**  Environmental restructuring  **Enablement**  **Restriction**  Using rules to reduce the opportunity to engage in the target behavior (or to increase the target behavior by reducing the opportunity to engage in competing behaviours | Guidelines  Regulation  Legislation  Communication/marketing  Fiscal measures  Environmental/social planning  Service provision |
| **Opportunity**  **Social opportunity**  Opportunity afforded by interpersonal influences, social cues and cultural norms that influence the way that we think about things, e.g. the words and concepts that make up our language | **Social influences**  Those interpersonal processes that can cause individuals to change their thoughts, feelings, or behaviours | **Restriction**  **Environmental** **restructuring**  **Modelling**  **Enablement** | Guidelines  Fiscal measures  Regulation  Legislation  Communication/marketing  Environmental/social planning  Service provision |
| **Motivation**  **Reflective motivation**    *Reflective processes involving plans (self-conscious intentions) and evaluations (beliefs about what is good and bad)* | **Social/professional role**  **and identity**  A coherent set of behaviours and displayed personal qualities of an individual in a social or work setting | **Education**  **Persuasion**  **Modelling** | Communication/marketing Guidelines  Regulation  Legislation  Service provision |
| **Motivation**  **Reflective motivation** | **Beliefs about capabilities**  Acceptance of the truth, reality, or validity about an  ability, talent, or facility that a person can put to constructive use | **Education**  **Persuasion**  **Modelling**  **Enablement** | Guidelines  Fiscal measures  Regulation  Legislation  Communication/marketing  Environmental/social planning  Service provision |
| **Motivation**  **Reflective motivation** | **Beliefs about consequences**  Acceptance of the truth,  reality, or validity about  outcomes of a behaviour  in a given situation) | **Education**  **Persuasion**  **Modelling** | Communication/marketing Guidelines  Regulation  Legislation  Service provision |
| **Motivation**  **Reflective motivation** | **Optimism**  The confidence that things  will happen for the best or  that desired goals will be  attained | **Education**  **Persuasion**  **Modelling**  **Enablement** | Guidelines  Fiscal measures  Regulation  Legislation  Communication/marketing  Environmental/social planning  Service provision |
| **Motivation**  **Reflective motivation** | **Goals**  Mental representations  of outcomes or end states  that an individual wants to achieve | **Education**  **Persuasion**  **Modelling**  **Enablement**  **Coercion**  Creating an expectation of punishment or cost  **Incentivisation**  Creating an expectation of reward | Guidelines  Fiscal measures  Regulation  Legislation  Communication/marketing  Environmental/social planning  Service provision |
| **Motivation**  **Reflective motivation** | **Intentions**  A conscious decision to  perform a behaviour or a  resolve to act in a certain  way | **Education**  **Persuasion**  **Modelling**  **Enablement**  **Coercion**  **Incentivisation** | Guidelines  Fiscal measures  Regulation  Legislation  Communication/marketing  Environmental/social planning  Service provision |
| **Motivation**  **Automatic motivation**  Automatic processes involving emotional reactions, desires (wants and needs), impulses, inhibitions, drive states and reflex responses | **Emotion**  A complex reaction pattern, involving experiential, behavioural, and physiological elements, by which the individual attempts to deal with a personally significant matter or event | **Persuasion**  **Modelling**  **Enablement**  **Coercion**  **Incentivisation** | Guidelines  Fiscal measures  Regulation  Legislation  Communication/marketing  Environmental/social planning  Service provision |
| **Motivation**  **Automatic motivation** | **Reinforcement**  Increasing the probability  of a response by arranging  a dependent relationship,  or contingency, between  the response and a given  stimulus | **Training**  **Incentivisation**  **Coercion**  **Environmental restructuring** | Guidelines  Fiscal measures Regulation  Legislation  Communication/marketing  Environmental/social planning  Service provision |
