# Supplementary material for: Barriers to and enablers of the use of the Otology Questionnaire Amsterdam in clinical practice—a qualitative post-implementation study
Source: J Patient Rep Outcomes. 2024 Aug 14;8:96. doi: 10.1186/s41687-024-00741-9 (PMC11324631; doi:10.1186/s41687-024-00741-9)
Supplement: Supplementary file 4 — Supplementary Material 4 [file 41687_2024_741_MOESM4_ESM.docx]

### **Appendix. Topics used in the focus groups and individual interviews**

## Focus group protocol: ENT professionals

**Q1. What are the first experiences or thoughts that come to mind when you think about using the OQUA in your consultation (or in clinical practice)?**

- To what extent are you familiar with the content and purpose of the OQUA?
- To what extent are you comfortable with how to access and use the OQUA in your consultation?

**Q2. What is the main reason (largest enabling factor) for you to do use the OQUA in your consultation?**

- In what ways could this facilitating factor be strengthened for you?
- What other (smaller) factors play a role for you in whether you do use the OQUA?
  (in terms of physical factors/social factors/environment/professional norms or identity)

**Q3. What is the main reason (largest barrier) for you not to use the OQUA in your consultation?**

- In what ways could this barrier be solved for you?
- What other (smaller) factors play a role for you in not using the OQUA?
  (in terms of physical factors/social factors/environment/professional norms or identity)

**Q4. What would motivate you to use the OQUA in your consultation?**

- What do you think might be the positive consequences of using the OQUA in your consultation?
- What do you think might be the negative consequences of using the OQUA in your consultation?

## Interview protocol: patients

*If the questionnaire was not completed by the patient, Q1 and Q2 can be skipped.*

**Q1. When you think (back) about completing the OQUA or its use in the consultation room by the ENT surgeon, what is your first experience or thought that comes to mind?**

- To what extent was it clear to you what the purpose of the OQUA was?
- To what extent was it clear to you where to find the OQUA? And how to complete the questionnaire?

**Q2. What was the main reason (largest enabling factor) for you to do complete the OQUA?**

- What other (smaller) factors played a role for you in whether you did complete the OQUA?
  (in terms of physical and social environment).

**Q3. Thinking back on receiving/completing the OQUA, were there any obstacles that made it difficult for you to complete the questionnaire?**

- What was the largest obstacle for you while completing the questionnaire/what was the main reason for you not completing the questionnaire?
- What other (smaller) obstacles did you experience while completing the questionnaire?
  (in terms of knowledge, skills, physical and social environment).

**Q4. What would motivate you to complete the questionnaire next time**?

- What did you experience as a positive or negative consequence of completing the OQUA/what do you think might be the positive or negative consequences of completing the OQUA?
- Do you have an idea or suggestion in which way we (ENT department of Amsterdam UMC) can support you in completing the questionnaire (again)?
