# Supplementary material for: Barriers to and enablers of the use of the Otology Questionnaire Amsterdam in clinical practice—a qualitative post-implementation study
Source: J Patient Rep Outcomes. 2024 Aug 14;8:96. doi: 10.1186/s41687-024-00741-9 (PMC11324631; doi:10.1186/s41687-024-00741-9)
Supplement: Supplementary file 5 — Supplementary Material 5 [file 41687_2024_741_MOESM5_ESM.docx]

## S5. Illustration of the coding process

The following is an excerpt from the focus group with ENT-professionals. The transcripts were coded according to four steps of Atkins' (2017) content analysis: (1) selecting and highlighting meaning units, (2) classifying the meaning units into categories of the COM-B model, (3) specifying the categories into the 14 domains of the TDF, (4) creating an overarching theme that provides a brief statement about the perceived barrier or enabler.

**COM-B:** Physical Opportunity

**TDF:** Environmental context and resources

**Theme:** Perceived (additional) time investment and administrative tasks to use the OQUA

**COM-B:** Reflective Motivation

**TDF:** Beliefs about consequences

**Theme:** Perceived obligation to review the results of the OQUA with the patient

*Respondent*: Mainly because I have not enough time in my consultation. I often feel quite overwhelmed in the outpatient clinic because of all the administrative tasks. It's not unwillingness, but you only have ten minutes with patient. When you think of all the things you already have to click on in your consultation in the electronic health record.... And I’m a little concerned that a questionnaire like this, for which the patient has taken some effort to complete, means that the patient wants to hear back that he has spent time to complete it. As a doctor, you then feel compelled to do something with it, even though your consultation time is running out and you just don't have time for it. Thereby, the way it is presented in the electronic health record, I cannot draw any conclusions from that. There are scores displayed, but no values. So, it is not sufficiently validated for me to use it in the consultation room. No, I can't do anything with it at the moment.

**COM-B:** Physical Capability

**TDF:** Skills

**Theme:** Lack of skills to interpret scores of the OQUA

**COM-B:** Reflective Motivation

**TDF:** Beliefs about consequences

**Theme:** Perceived unclarity of the clinical significance of the OQUA
